# Factors Influencing Mental Health Among Chinese Medical and Non-medical Students in the Early Stage of the COVID-19 Pandemic

**DOI:** 10.3389/fpubh.2021.603331

**Published:** 2021-05-20

**Authors:** Peng Xiong, Wai-kit Ming, Caiyun Zhang, Jian Bai, Chaohua Luo, Wenyuan Cao, Fan Zhang, Qian Tao

**Affiliations:** ^1^Department of Public Health and Preventive Medicine, School of Medicine, Jinan University, Guangzhou, China; ^2^Division of Medical Psychology and Behavior Science, School of Medicine, Jinan University, Guangzhou, China; ^3^School of Medicine, Jinan University, Guangzhou, China; ^4^The Department of Health and Physical Education, The Education University of Hong Kong, Hong Kong, China

**Keywords:** anxiety, depression, stress, pandemic of COVID-19, university students

## Abstract

**Background:** The 2019 coronavirus disease (COVID-19) pandemic is a public health emergency of international concern. This study aimed to assess the psychological outcomes and their influencing factors among medical and non-medical University students during the COVID-19 pandemic in China.

**Methods:** A cross-sectional online survey using structured questionnaires was conducted from February 20 to March 20, 2020. Psychological outcomes were assessed according to the Depression, Anxiety, and Stress Scale (DASS-21). Influencing factors were assessed by COVID-19 knowledge, mindful coping scale, and sense of control scale.

**Results:** Our sample is comprised of 563 University students (male = 172, mean age = 21.52). Among them, 382 are medical students. Among the participants, 12.26, 18.47, and 8.53% have moderate to severe levels of depression, anxiety, and stress symptoms, respectively. Compared with the non-medical students, the medical students had a higher knowledge level of COVID-19, a higher sense of awareness, and fewer mental health symptoms. After controlling the covariance, perceived constraints of sense of control were negatively associated with depression, anxiety, and stress among both medical and non-medical students. Prevention of negative emotions by mindful coping was negatively associated with depression and anxiety among non-medical students. Knowledge of COVID-19 is not associated with mental distress among medical and non-medical students.

**Conclusions:** During the COVID-19 pandemic in China, the mental health of University students was affected. Our findings suggested that a sense of control is a protective factor for both medical and non-medical students, while mindful coping is a protective factor for only non-medical students.

## Background

An outbreak of novel coronavirus disease-19 (COVID-19) infection is spreading internationally (1). On January 30, 2020, the World Health Organization (WHO) declared the global COVID-19 outbreak a public health emergency of international concern (2). According to the statistics released by the WHO, there have been over 109,000,000 confirmed cases of COVID-19 infection worldwide, with at least 2,400,000 deaths as of February 17, 2021 (3). To efficiently cope with the COVID-19 pandemic, the Chinese government implemented rapid and comprehensive public health emergency interventions. For instance, schools and other public places were closed, and students were encouraged to stay at home.

The COVID-19 pandemic has a negative impact not only on physical health but also on psychological well-being, particularly when the outbreak lasts longer and is still developing. The psychological consequences of COVID-19 have been reported to include depressed mood, anxiety, poor sleep, and increased stress level (4). The COVID-19 pandemic, with its rapid spread and high mortality, constitutes a unique case of an acute, large-scale, and uncontrollable stressor. It is well-established that stress can have a significant effect on individuals' psychological well-being, particularly when the individual cannot cope with the stress (5). Enough knowledge and concrete information about COVID-19 would help to better cope with it. A wide range of medical knowledge was involved in this newly emerging disease, such as the clinical presentations of coronavirus pneumonia, its pathogenesis, current transmission status, and prevention and control methods. We expected that students with more knowledge on COVID-19 would be able to better cope with it.

Mindfulness was widely recognized as an effective treatment for psychological and somatic symptoms (6). Coping has been defined as “constantly changing cognitive and behavioral efforts to manage specific external and/or internal demands that are appraised as taxing or exceeding the resources of the person” (7), which is one of the determinants in how individuals would react to major stressors such as the pandemic of COVID-19. Previous literature has found that mindful coping is a protective factor when dealing with stressful events (8). Accumulating evidence has suggested that mindful coping effectively reduces stress and anxiety in college students (9). Hence, we use the four sectors to provide the foundation of mindful coping: awareness, distraction, preventing negative emotions, and constructive self-assertion. Facing this critical situation, students with a higher level of mindful coping are expected to better cope with the pandemic of COVID-19.

Another potential factor influencing psychological outcomes under the effects of the COVID-19 pandemic is a sense of control. Sense of control has been described as a cognitive style that attributes life outcomes to personal behavior and abilities or to external forces such as powerful others, chance, or luck (10). A sense of personal control is a learned, universal belief that people can and do master, control, and shape their lives. Having a high sense of control is related to positive psychological outcomes and proactive behaviors with linking to the ability of action prevention and healthy feeling, whereas an impaired sense of control is associated with depression and anxiety (11, 12). With regard to University students, a higher sense of control is related to better college adjustment among first-year women students (13) and a higher grade point average (GPA) (14). When dealing with the COVID-19 outbreak, students with a higher sense of control are expected to be better able to cope with mental distress.

The COVID-19 pandemic is a global challenge in higher education, especially in medical education. Studies compared with peers in the general population and with non-medical college students before the COVID-19 pandemic showed the prevalence of mental distress in medical students was higher than in others (15–17). Medical students were thought to be professionally trained and have more knowledge of causes, prevention, and treatment of diseases than others. There was not much known about COVID-19 during the early stages of the pandemic. They were not sure if their historical knowledge and skill could manage their fear of this emerging epidemic. Medical study is different from that of other college students as it includes high-intensity bedside teaching and practice. However, clinical training and clerkships were transitioned to online learning during the pandemic. A study conducted during the MERS-COV outbreak showed that over 20% of medical students reported moderate or severe anxiety levels (18). Prevention of negative emotions is important.

Given the relationship identified in the literature between the mental health and mindful coping, and sense of control, the present study performed a web-based cross-sectional study to assess the psychological impact of the COVID-19 pandemic on medical students and to compare their responses with non-medical students. We hypothesize that medical students experienced higher levels of mental distress than non-medical students; the knowledge of COVID-19, mindful coping, and a sense of control are protective factors on mental health when students face the COVID-19 pandemic.

## Methods

### Setting and Sample

A cross-sectional survey using structured questionnaires was conducted among University students at Jinan University in Guangzhou, Guangdong, China. The data was collected from February 20 to March 20, 2020. During this period, most areas in China were still under the influence of the COVID-19 pandemic. Given the situation, online questionnaires (wjx.cn, which is one of the most popular online survey platforms in China) were distributed to University students via WeChat groups and WhatsApp messaging application with snowball sampling method. A total of 563 participants completed the survey. The study was approved by the ethics committee of Jinan University, and participants provided e-written consents before they answer the questions. After completion of the questionnaire, participants would have a 10% chance to win an RMB 20 coupon in a lucky draw.

### Instruments

The questionnaires consisted of COVID-19 knowledge, mindful coping, sense of control, and mental health. Participants' demographic information was collected, including age, gender, grade, education level, academic major, residence location during the pandemic, and whether confirmed or suspected patients were around.

#### COVID-19 Knowledge

The knowledge questionnaire were developed based on the Diagnosis and Treatment of COVID-19, National Health Commission, the People's Republic of China (http://www.gov.cn/zhengce/zhengceku/2020-02/19/content_5480948.htm). In total, there were 23 true/false questions, including two questions regarding the characteristics of coronavirus (e.g., coronavirus is sensitive to heat and UV irradiation), four questions regarding transmission route (e.g., the “silent carriers” who showed no symptoms may also pass the virus to others), four regarding clinical presence (e.g., the light symptoms of COVID-19 include fever and fatigue, and may not show the symptoms of pneumonia), six regarding diagnosis and treatment (e.g., no effective pharmacological treatment for COVID-19 has been developed), and seven questions regarding preventative methods (e.g., using 75% alcohol could be an effective agent against the virus). For each question, participants were asked to respond on a true/false/I don't know basis. A total score, ranging from 0 to 23, was obtained by counting the total number of correct answers, with a higher score indicating a higher level of COVID-19 knowledge. The Cronbach alpha in our sample was 0.76, indicating good reliability.

#### Sense of Control

The Chinese version of a 12-item sense of control scale was adopted (19). It includes two subscales: the personal mastery subscale assesses how people feel they can control themselves (e.g., whether I can get what I want with my own hands), and the perceived constraint subscale evaluates how people feel they cannot control the outcome (e.g., I often feel helpless in dealing with the problems of life). Participants were asked to rate on a 7-point Likert scale (1 = *strongly agree*, 7 = *strongly disagree*). After reversely coding the items in the personal mastery subscale, the average score of all items was obtained, with a higher score suggesting a greater sense of control. A previous study in a Chinese sample suggested good reliability and validity of the scale (20), and in our sample, a Cronbach alpha was 0.77, 0.82, and 0.85 for the personal mastery subscale, the perceived constraint subscale, and the whole scale, respectively.

#### Mindful Coping

The mindful coping scale developed by Tharaldsen et al. was adopted in our study (6). The questionnaire was translated from English to Chinese by an author (FZ), and then it was back-translated by two authors (QT and PX) independently. The scale consisted of 23 items, evaluating an individual's level of awareness, distraction, negative emotions prevention, and constructive self-assertion. With a 5-point Likert scale (1 = *never/hardly ever*, 5 = *always*), participants were asked to choose how they will react when facing difficulties. Total scores of the whole set and four subscales were generated, with a higher score indicating a higher level of mindful coping. In our sample, the Cronbach alpha was 0.74, 0.82, 0.77, 0.89, and 0.89 for the awareness subscale, distraction subscale, negative emotions prevention subscale, constructive self-assertion subscale, and the whole scale, respectively. It suggested good reliability of the Chinese version.

#### Mental Health

The individuals' mental health was assessed by the Chinese version of the 21-item Depression Anxiety Stress Scale (DASS-21) (21). The DASS-21 is a well-developed instrument evaluating an individual's level of depression, anxiety, and stress, with seven items in each subscale. Using a four-point Likert scale, it asked participants whether the described situation applies to them by choosing from 0 “*did not apply to me at all*” to 3 “*applied to me very much*.” The total score of each subscale was multiplied by two and ranged from 0 to 42. A higher score means a more severe level of depression, anxiety, and stress. The total score 0–13, 0–9, 0–18 of the subscale means a normal to mild level of depression, anxiety, and stress, respectively. A total score equal to and higher than 14, 10, 19 means the moderate to severe level of depression, anxiety, and stress, respectively (22). In our sample, the Cronbach alpha was 0.81, 0.80, and 0.87 for the depression subscale, anxiety subscale, and stress subscale, respectively. It suggested an excellent reliability.

### Data Analysis

Descriptive statistics were analyzed for the demographic variables and scores of questionnaires and scales. Continuous variables were described with mean and standard deviation, and the categorical variables were described with cases (*n*) and percentage (%). Independent two-sample *t*-tests to compare continuous variables and chi-square tests for categorical variables were conducted between medical and non-medical students, respectively. Pearson correlation was conducted. Linear regression was performed to estimate the associations between knowledge, sense of control, mindful coping, and mental health status. Univariate analyses were used in model 2. Model 2 was adjusted for all the demographic variables. All the statistical analysis was performed with STATA software 14.2 (STATA Corp., TX, US). *P*-value < 0.05 with two tails was considered to be statistically significant.

## Results

### Participants Characteristics

A total of 563 University students were recruited. Among them, 382 are medical students, and 181 are from other majors such as economics, law, education, and history. The average age was 21.52 (2.50) years old, and the majority (83.36%) were undergraduate students. Of this total, 12.26, 18.47, and 8.53% of students have moderate to severe levels of depression, anxiety, and stress symptoms, respectively. The percentage of those having moderate to severe mental symptoms is higher among non-medical students than medical students (see Table 1).

**Table 1:** Characteristics of the participants.

**Items**	**All *n* (%)/mean ± SD**	**Medical students *n* (%)/mean ± SD**	**Non-medical students *n* (%)/mean ± SD**	***P*-value**
**Gender**				
Male	172 (30.55)	126 (32.98)	46 (25.41)	0.069
Female	391 (69.45)	256 (67.02)	135 (74.59)	
Age	21.52 ± 2.50	21.34 ± 2.43	21.90 ± 2.59	0.324
**Education level**				
Bachelor	456 (83.36)	322 (85.41)	134 (78.82)	0.012^*^
Master	83 (15.17)	53 (14.06)	30 (17.65)	
Doctoral	8 (1.46)	2 (0.53)	6 (3.53)	
**Residence location during the COVID-19 pandemic**				
Hubei Province	9 (1.60)	5 (1.31)	4 (2.21)	0.109
Mainland China (Except Hubei)	505 (89.70)	342 (89.53)	163 (90.06)	
Hong Kong SAR, China	20 (3.55)	12 (3.14)	8 (4.42)	
Macao SAR, China	13 (2.31)	13 (3.40)	0 (0.00)	
Foreign countries	16 (2.84)	10 (2.62)	6 (3.31)	
**Any confirmed/suspected patients with COVID-19 around you**				
Yes	10 (1.78)	4 (1.05)	6 (3.31)	0.057
No	553 (98.22)	378 (98.95)	175 (96.69)	
**Depression**				
Normal to mild	494 (87.74)	341 (89.27)	153 (84.53)	0.109
Moderate to severe	69 (12.26)	41 (10.73)	28 (15.47)	
**Anxiety**				
Normal to mild	459 (81.53)	324 (84.82)	135 (74.59)	0.003^**^
Moderate to severe	104 (18.47)	58 (15.18)	46 (25.41)	
**Stress**				
Normal to mild	515 (91.47)	359 (93.98)	156 (86.19)	0.002^**^
Moderate to severe	48 (8.53)	23 (6.02)	25 (13.81)	

* *p < 0.05;*.

** *p < 0.01*.

### Average Scores and Associations of Measurements Among Medical and Non-medical Students

There was a statistical significance of scores on knowledge of COVID-19, awareness subscale of mindful coping scale, and three dimensions of DASS-21. The results indicated that the medical students had a higher level of knowledge, a higher sense of awareness, and fewer mental health symptoms (see Table 2).

**Table 2:** Average scores of knowledge, mindful coping, sense of control, and mental health among participants.

**Items**	**Medical students (mean ± SD)**	**Non-medical students (mean ± SD)**	***t*-score**	***p***
Knowledge of COVID-19	15.10 ± 3.36	13.29 ± 3.81	32.78	< 0.01^**^
Total score of sense of control	4.41 ± 0.78	4.28 ± 0.74	3.28	0.51
Score of personal mastery subscale	5.06 ± 0.90	4.94 ± 0.91	2.13	0.88
Score of perceived constraints subscale	4.08 ± 0.90	3.95 ± 0.83	2.63	0.27
Total score of mindful coping scale	78.31 ± 11.23	76.52 ± 11.78	3.03	0.08
Score of awareness subscale	19.88 ± 3.46	19.06 ± 3.24	7.22	0.01^*^
Score of distraction subscale	23.30 ± 4.85	23.22 ± 4.79	0.03	0.86
Score of preventing negative emotions subscale	17.08 ± 3.39	16.55 ± 3.59	2.88	0.09
Score of constructive self-assertion subscale	18.06 ± 3.36	17.70 ± 3.55	1.39	0.24
Score of depression	2.34 ± 3.13	3.23 ± 3.49	9.20	0.003^**^
Score of anxiety	2.30 ± 3.03	2.96 ± 3.32	5.44	0.02^*^
Score of stress	2.78 ± 3.59	4.13 ± 4.27	15.32	< 0.01^**^

* *p < 0.05*;

** *p < 0.01*.

### Correlation Results Among Medical and Non-medical Students

Among the medical students, the knowledge score was significantly correlated with the total scores of sense of control (*r* = 0.13), personal mastery subscale (*r* = 0.17), and total score of mindful coping scale (*r* = 0.20) and its four subscales, respectively. Total score of sense of control was significantly correlated with scores of depression (*r* = −0.46), anxiety (*r* = −0.43), and stress (*r* = −0.44). Total score of mindful coping scale was also significantly correlated with scores of depression (*r* = −0.21), anxiety (*r* = −0.18), and stress (*r* = −0.20) (see Table 3). Among the non-medical students, the knowledge score was significantly correlated with the total score of sense of control (*r* = 0.24), personal mastery subscale (*r* = 0.23), perceived constrains subscale (*r* = 0.20), but not other scales. Total score for sense of control was significantly correlated with scores of depression (*r* = −0.50), anxiety (*r* = −0.43), and stress (*r* = −0.43). Total score for mindful coping scale was also significantly correlated with scores of depression (*r* = −0.25), anxiety (*r* = −0.22), and stress (*r* = −0.15) (Table 4).

**Table 3:** The associations between knowledge and sense of control, mindful coping, and mental health among medical students.

**Items**	**1**	**2**	**3**	**4**	**5**	**6**	**7**	**8**	**9**	**10**	**11**	**12**
1. Knowledge of COVID-19	1											
2. Total score of sense of control	0.13^*^	1										
3. Score of personal mastery subscale	0.17^**^	0.72^**^	1									
4. Score of perceived constraints subscale	0.08	0.94^**^	0.44^**^	1								
5. Total score of mindful coping scale	0.20^**^	0.46^**^	0.49^**^	0.34^**^	1							
6. Score of awareness subscale	0.13^*^	0.28^**^	0.32^**^	0.21^**^	0.71^**^	1						
7. Score of distraction subscale	0.15^**^	0.32^**^	0.34^**^	0.25^**^	0.81^**^	0.39^**^	1					
8. Score of preventing negative emotions subscale	0.21^**^	0.47^**^	0.51^**^	0.35^**^	0.77^**^	0.39^**^	0.55^**^	1				
9. Score of constructive self-assertion subscale	0.11^*^	0.30^**^	0.32^**^	0.22^*^	0.67^**^	0.40^**^	0.32^**^	0.36^**^	1			
10. Score of depression	−0.003	−0.46^**^	−0.30^**^	−0.45^**^	−0.21^**^	−0.10	−0.17^**^	−0.27^**^	−0.08	1		
11. Score of anxiety	−0.01	−0.43^**^	−0.25^**^	−0.43^**^	−0.18^**^	−0.16^**^	−0.06	−0.20^**^	−0.16^**^	0.72^**^	1	
12. Score of stress	−0.01	−0.44^**^	−0.26^**^	−0.44^**^	−0.20^**^	−0.16^**^	−0.09	−0.19^**^	−0.16^**^	0.76^**^	0.83^**^	1

* *p < 0.05*;

** *p < 0.01*.

**Table 4:** The associations between knowledge and sense of control, mindful coping, and mental health among non-medical students.

**Items**	**1**	**2**	**3**	**4**	**5**	**6**	**7**	**8**	**9**	**10**	**11**	**12**
1. Knowledge of COVID-19	1											
2. Total score of sense of control	0.24^**^	1										
3. Score of personal mastery subscale	0.23^**^	0.75^**^	1									
4. Score of perceived constraints subscale	0.20^**^	0.93^**^	0.45^**^	1								
5. Total score of mindful coping scale	0.09	0.38^**^	0.41^**^	0.28^**^	1							
6. Score of awareness subscale	0.12	0.18^**^	0.21^**^	0.13	0.61^**^	1						
7. Score of distraction subscale	0.07	0.27^**^	0.34^**^	0.17^*^	0.87^**^	0.35^**^	1					
8. Score of preventing negative emotions subscale	0.04	0.41^**^	0.43^**^	0.31^**^	0.81^**^	0.29^**^	0.67^**^	1				
9. Score of constructive self-assertion subscale	0.07	0.32^**^	0.28^**^	0.27^**^	0.77^**^	0.36^**^	0.54^**^	0.50^**^	1			
10. Score of depression	−0.01	−0.50^**^	−0.34^**^	−0.48^**^	−0.25^**^	−0.15^*^	−0.14	−0.35^**^	−0.16^*^	1		
11. Score of anxiety	−0.05	−0.43^**^	−0.25^**^	−0.34^**^	−0.22^**^	−0.21^**^	−0.10	−0.27^**^	−0.14	0.83^**^	1	
12. Score of stress	−0.04	−0.43^**^	−0.23^**^	−0.46^**^	−0.15^*^	−0.16^*^	−0.04	−0.26^**^	−0.04	0.84^**^	0.82^**^	1

* *p < 0.05*;

** *p < 0.01*.

### Regression Results Among Medical and Non-medical Students

In regression model 1, among medical and non-medical students, perceived constraints of sense of control were significantly associated with depression, anxiety, and stress symptoms, respectively. The significant results remained the same after controlling for the demographic variables in model 2. The distraction subscale of mindful coping was significantly associated with anxiety among medical students. Negative emotion prevention of mindful coping was significantly associated with depression and anxiety among non-medical students. Both awareness and negative emotions prevention of mindful coping were significantly associated with anxiety and stress among non-medical students. Knowledge is not associated with mental distress among medical and non-medical students (see Tables 5, 6).

**Table 5:** Regression model amongmedical students.

	**Items**	**Depression**				**Anxiety**				**Stress**			
		**Coef**.	**Beta**	***t***	***p***	**Coef**.	**Beta**	***t***	***p***	**Coef**.	**Beta**	***t***	***p***
Model 1	Knowledge	0.06	0.04	1.35	0.18	0.06	0.64	1.30	0.19	0.04	0.04	0.08	0.42
	Personal mastery subscale	−0.10	0.24	−1.55	0.12	−0.18	−0.05	−0.73	0.47	−0.31	−0.07	−1.20	0.23
	Perceived constraints subscale	−0.38	0.21	−6.46	< 0.01^**^	−1.33	−0.39	−6.43	< 0.01^**^	−1.58	−0.40	−7.21	< 0.01^**^
	Awareness subscale	0.03	0.05	0.60	0.55	−0.07	−0.08	−1.25	0.21	−0.07	−0.07	−1.16	0.25
	Distraction subscale	−0.02	0.04	−0.27	0.79	0.09	0.14	2.25	0.03^*^	0.05	0.07	1.32	0.19
	Preventing negative emotions subscale	−0.12	0.06	−1.79	0.08	−0.07	−0.08	−0.97	0.33	−0.02	−0.02	−0.23	0.82
	Self–assertion subscale	0.06	0.05	1.25	0.21	−0.05	−0.05	−0.88	0.38	−0.04	−0.04	−0.70	0.49
Model 2	Knowledge	0.04	0.05	0.89	0.37	0.05	0.06	1.10	0.27	0.03	0.03	0.64	0.52
	Personal mastery subscale	−0.34	−0.10	−1.36	0.17	−0.11	−0.03	−0.44	0.66	−2.30	−0.07	−1.15	0.25
	Perceived constraints subscale	−1.36	−0.39	−6.43	< 0.01^**^	−1.38	−0.42	−7.01	< 0.01^**^	−1.62	−0.42	−7.79	< 0.01^**^
	Awareness subscale	0.03	0.03	0.60	0.55	−0.06	−0.07	−1.19	0.23	−0.07	−0.07	−1.15	0.25
	Distraction subscale	−0.02	−0.03	−0.41	0.68	0.86	0.14	2.23	0.02^*^	0.05	0.07	1.17	0.24
	Preventing negative emotions subscale	−0.11	−0.12	−1.69	0.09	−0.07	−0.08	−0.98	0.33	−0.03	−0.03	−0.36	0.72
	Self-assertion subscale	0.09	0.09	1.59	0.11	−0.02	−0.03	−0.42	0.67	0.007	−0.01	0.11	0.91

*Model 1 means the rude regression; Model 2 means the regression after control the demographic variables*.

* *p < 0.05*;

** *p < 0.01*.

**Table 6:** Regression model among non-medical students.

	**Items**	**Depression**				**Anxiety**				**Stress**			
		**Coef**.	**Beta**	***t***	***p***	**Coef**.	**Beta**	***t***	***p***	**Coef**.	**Beta**	***t***	***p***
Model 1	Knowledge	0.10	0.06	1.61	0.11	0.04	0.05	0.81	0.42	0.05	0.09	0.77	0.44
	Personal mastery subscale	−0.11	0.31	−1.35	0.18	−0.09	−0.02	−0.29	0.77	0.02	0.003	0.05	0.96
	Perceived constraints subscale	−0.39	0.33	−4.86	< 0.01^**^	−1.57	−0.40	−4.75	< 0.01^**^	−2.23	−0.44	−5.59	< 0.01^**^
	Awareness subscale	−0.07	0.07	−1.11	0.27	−0.18	−0.17	−2.50	0.01^*^	−0.20	−0.15	−2.21	0.03^*^
	Distraction subscale	0.15	0.06	1.72	0.09	0.10	0.15	1.78	0.07	0.17	0.19	2.32	0.02^*^
	Preventing negative emotions subscale	−0.30	0.09	−3.19	< 0.002^**^	−0.20	−0.21	−2.26	0.03^*^	−0.34	−0.29	−3.45	< 0.001^**^
	Self-assertion subscale	0.06	0.09	0.68	0.50	0.01	0.01	0.70	0.48	0.21	0.18	2.19	0.03^*^
Model 2	Knowledge	0.10	0.11	1.70	0.09	0.04	0.04	0.62	0.53	0.07	0.06	0.93	0.36
	Personal mastery subscale	−0.49	−1.22	−1.35	0.18	−0.06	−0.02	−0.18	0.52	0.00	0.00	0.00	1.00
	Perceived constraints subscale	−1.51	−0.36	−4.58	< 0.01^**^	−1.67	−0.41	−4.66	0.86	−2.20	−0.42	−5.10	< 0.01^**^
	Awareness subscale	−0.09	−0.08	−1.13	0.26	−0.16	−0.15	−2.15	< 0.01^**^	−0.19	−0.14	−1.95	0.054
	Distraction subscale	0.11	0.14	1.54	0.13	0.09	0.13	1.41	0.16	0.15	0.17	1.97	0.05
	Preventing negative emotions subscale	−0.33	−0.33	−3.48	< 0.001^**^	−0.23	−0.25	2.80	0.006^**^	−0.41	−0.34	−3.94	< 0.01^**^
	Self-assertion subscale	0.13	0.13	1.36	0.18	0.14	0.14	1.76	0.08	0.28	0.22	2.67	0.008^**^

*Model 1 means the rude regression; Model 2 means the regression after control the demographic variables*.

* *p < 0.05*;

** *p < 0.01*.

## Discussion

By conducting a web-based survey among 563 University students, the current study investigated the psychological effects of the COVID-19 pandemic on University students' mental health and examined the roles of knowledge about the pandemic, mindful coping, as well as sense of control. The findings showed that both mindful coping and a sense of control would benefit mental health for both medical and non-medical students. However, the effects of specific constructs in mindful coping and sense of control varied between medical and non-medical students.

Our study revealed that non-medical students reported higher prevalence of moderate to severe levels of depression, anxiety, and stress symptoms than medical students. This was in line with the similar study conducted by Xie et al. (23). During the pandemic of COVID-19, previous related studies showed that 22.4–35.5% medical students reported depressive symptoms (24, 25) and 22.1–28% reported anxiety symptoms (24, 26). Among general University students, depressive symptoms ranged from 12.2 to 56.8% (27, 28), and anxiety symptoms ranged from 7.7 to 15.43% (27, 29). An online mental survey targeting different populations showed the prevalence of depression, anxiety, and stress-related symptoms was 50.7, 44.7, and 73.4%, respectively (30). The prevalence among the general population was much higher than University students, and this might be due to the higher perceived knowledge level among University students. For the general population, they obtained information from various channels and lacked ability to distinguish accurate information. It is possible that the public fear of COVID-19 at the beginning of the outburst was driven by the fear of the unknown (31).

Despite that medical students showed a higher level of knowledge about COVID-19 than non-medical students, knowledge did not exert any significant effect on the mental health of University students. At first glance, the result seemed inconsistent with our hypothesis and several recent findings. For instance, a study reported that a greater level of knowledge was associated with a more optimistic attitude and better preventive practice in a larger sample (32). It is possible that the effect of knowledge is more important at the behavioral and attitude levels when predicting mental states, such as depression, anxiety, and stress. The previous study suggested that more knowledge about COVID-19 might help reduce anxiety and depression, but it must be directed to the promotion of health behaviors and to the recognition of fake news (9). The role of knowledge could be reduced or even silenced by other psychological constructs, such as coping and sense of control. One study conducted through two large-scale nationwide surveys in China revealed that participants with higher perceived knowledge about COVID-19 but not the actual knowledge was positively associated with sense of control, which in turn protected their emotional well-being during the outbreak (33). In fact, we have further tested the mediation role of sense of control in the relationship between knowledge and mental health among medical students and non-medical students, respectively. The results showed that only among non-medical students, perceived constraint has mediated the effects of knowledge on mental health, such that greater knowledge was associated with lower mental distress via reduced perceived constraint. It is possible that for non-medical students, knowing more about the pandemic would help reduce the helpless feelings with the outcome; however, this effect was not found among medical students who may be habituated with this type of knowledge and need other novel resources to cope.

In line with existing literature, a positive association between a sense of control and mental health was found among all students. A previous study investigating the relationship between sense of control and depression has found that across 23 countries, a higher sense of control was associated with fewer depressive symptoms among college students (34). This protective effect could be because a sense of control may prompt individuals to locate attention to solutions and take actions in stressful situations (35). In other words, people with a higher sense of control might be better prepared for upcoming stressors and find them less unbearable. Sense of control could be linked to *stable influences*, such as a high sense of personal control would be the important determinant of coping behaviors (36). Therefore, people with a higher sense of control displayed greater resilience during the COVID-19 pandemic and developed fewer symptoms of depression, anxiety, and stress.

Mindful coping, as a specific type of adaptive coping in facing stress, was found to primarily benefit non-medical students. In particular, among non-medical students, greater awareness about the situation was related to lower anxiety, and preventing negative emotions could lead to reduced anxiety, depression, and stress. However, among medical students, no effect was found of awareness and negative emotion prevention, while distraction showed a small but significant effect opposite to our hypothesis. A higher level of distraction (i.e., engaging in different senses such as looking at something beautiful or listening to something enjoyable) was associated with greater anxiety. This finding extended the existing evidence for the benefit of mindful coping and identified that awareness about a situation and preventing negative emotions seems more effective when coping with stress. The negative effect of distraction for medical students could be consistent with a previous study that found medical students have greater difficulties in connecting with the body or sensations, as their anatomic knowledge could intrude when trying to engage in the senses (37). Meanwhile, it is also possible that they would “think about” the physiological reaction in negative ways instead of directly experiencing it, thus eliminating the relaxation effect of mindfulness. However, it is noteworthy that medical students showed better mental condition in facing COVID-19, i.e., lower depression, anxiety, and stress. This could be a result of their stressful training in daily life, which helps them become more habituated to stressful situations and less susceptible to the effects of COVID-19 and its related consequences.

Our findings have provided an updated profile of medical and non-medical students' knowledge, coping, and psychological outcomes under the influence of the COVID-19 pandemic and addressed how mindful coping and a sense of control may benefit an individual's mental health. However, there are a few limitations that should be acknowledged. First, as mentioned above, the effects of knowledge and coping may function at different levels, e.g., attitude, behavioral, or cognitive levels. By adopting more comprehensive assessments of outcomes, a clearer picture might be obtained of how University students face the contextual stress caused by the COVID-19 pandemic. Second, given that the outburst of COVID-19 is a recent situation, it is not possible to collect longitudinal data, which prevents us from understanding the effect of mindful coping and sense of control in the long run. Moreover, based on our findings, experiments, and intervention studies, manipulating mindful coping and sense of control should be conducted to further explore the most effective ways of helping University students maintain better psychological health amid a pandemic like COVID-19.

## Conclusion

To understand the coping strategies and mental status among medical and non-medical students, we have examined the roles of COVID-19 related knowledge, sense of control, and mindful coping in mental health. Surprisingly, no significant effect was found for knowledge. Meanwhile, sense of control was found to benefit both groups of students, while mindful coping, especially awareness about the situation and preventing negative emotions, was more beneficial for non-medical students. Therefore, to maintain the mental health of University students, support programs should take sense of control and mindful coping into consideration. Moreover, because non-medical students showed more symptoms of anxiety and depression in the pandemic, mindful-based intervention and programs to increase their sense of control should be provided.

## Data Availability Statement

The raw data supporting the conclusions of this article will be made available by the authors, without undue reservation.

## Ethics Statement

The studies involving human participants were reviewed and approved by ethics committee of Jinan University. The patients/participants provided their written informed consent to participate in this study.

## Author Contributions

PX contributed to data collection, data analysis, discussion on results, writing, and preparation of the manuscript. W-kM and CZ contributed to data collection, data analyses, writing, and preparation of the manuscript. CL, JB, and WC contributed to data collection and data analyses. FZ and QT contributed to study design, data collection, results interpretation, discussion of results, writing, and preparation of the manuscript. All authors read and approved the final manuscript.

## Conflict of Interest

The authors declare that the research was conducted in the absence of any commercial or financial relationships that could be construed as a potential conflict of interest.

## Abbreviations

COVID-19: Novel coronavirus disease 2019
GPA: grade point average
DASS-21: 21-item Depression Anxiety Stress Scale.
